# Disentangling the PIGD classification for the prediction of cognitive impairment in de novo Parkinson’s disease

**DOI:** 10.1007/s00415-021-10730-3

**Published:** 2021-08-02

**Authors:** Daniele Urso, Valentina Leta, Lucia Batzu, Tayyabah Yousaf, Chloe Farrell, Daniel J. van Wamelen, K. Ray Chaudhuri

**Affiliations:** 1 Department of Neurosciences, Institute of Psychiatry, Psychology and Neuroscience, King’s College London, London, UK; 2 Parkinson’s Foundation Centre of Excellence, King’s College Hospital, Denmark Hill, London, UK; 3 Department of Clinical Research in Neurology, Center for Neurodegenerative Diseases and the Aging Brain, University of Bari ‘Aldo Moro’, “Pia Fondazione Cardinale G. Panico”, Tricase, Lecce Italy; 4 Department of Neurology, Donders Institute for Brain, Cognition and Behaviour, Radboud University Medical Center, Nijmegen, The Netherlands

**Keywords:** Parkinson’s disease, Cognitive impairment, Postural instability, PIGD

## Abstract

**Background:**

Postural Instability and Gait difficulties (PIGD) subtype has been associated with worse cognitive performance in Parkinson’s disease (PD).

**Objective:**

To investigate whether PIGD subtype classification or PIGD-related clinical features predict the development of cognitive decline in de novo PD patients.

**Methods:**

Data from 422 PD patients with de novo PD were obtained from the PPMI database. At follow-up (up to 6 years), patients were categorized as having cognitive impairment or not. Multivariate Cox survival analysis was carried out including motor subtype and individual MDS-UPDRS items defining PIGD phenotype as predictors. Previously validated clinical predictors of cognitive impairment were included in the model as covariates. Occurrence of cognitive impairment at follow-up was used as the time-to-event and Kaplan–Meier curve was generated.

**Results:**

At baseline, 76 patients were classified as PIGD, 299 tremor-dominant and 47 as indeterminate. Development of cognitive impairment was not associated with PIGD subtype (*p* = 0.252). When individual MDS-UPDRS items were interrogated in the model, postural instability proved to be an independent predictor of cognitive impairment (HR = 2.045; 95%CI: 1.068–3.918; *p* = 0.031), while gait difficulties were not associated with cognitive decline (*p* = 0.870).

**Conclusions:**

Our findings suggest that postural instability, as assessed by MDS-UPDRS III, may serve as a possible indicator of the risk of developing cognitive impairment in de novo PD patients rather than the PIGD phenotype.

## Introduction

Postural instability is a cardinal feature of Parkinson’s disease (PD), specifically in advanced motor stages [1]. Together with gait difficulties and freezing, it defines the motor subtype called ‘Postural Instability and Gait difficulties (PIGD)’ that has less favourable motor and non-motor outcomes and then the tremor-dominant (TD) subtype [2–4]. The classification in PIGD and TD subtypes is based on items from the Unified Parkinson’s Disease Rating Scale (UPDRS) [3, 5]. Compared with patients with the TD subtype, those with PIGD subtype are typically older, less responsive to levodopa and are more likely to develop motor fluctuations and dyskinesia, as well as exhibit a greater burden of non-motor symptoms and faster disease progression [6–8]. The PIGD phenotype has also been associated with worse cognitive performance in PD [3, 7, 9–11]. Pathological, neuroimaging, and biochemical evidence has justified the biological validity of this subtyping [12], with differences in amyloid-β distribution patterns potentially explaining the relationship between PIGD and cognitive impairment [13, 14].

However, some controversies exist on this topic, since many studies claimed no differences in long-term outcomes between these subtypes [15–18]. This variability can be explained given the instability of the classification of PD versus PIGD with patients shifting between phenotypic subtypes as the disease progresses [19], and approximately 20% of patients remaining unclassified as part of an intermediate subgroup [20]. In this respect, there have been some attempts to discriminate various phenotypes within the PIGD continuum [21].

Therefore, in this study, we aim to investigate whether PIGD phenotype classification or specific PIGD-related clinical features could predict the development of cognitive impairment after 6-year follow-up in a cohort of de novo PD patients.

## Methods

### Participants

We included 422 de novo PD patients from the Parkinson’s Progression Markers Initiative (PPMI) database. The PPMI is an ongoing prospective, observational, international, multicentre study aimed at identifying clinical biomarkers of PD in a large cohort of participants with early PD at enrolment [22]. The aims and methodology of the study have been extensively published elsewhere and are available at www.ppmi-info.org/study-design. Inclusion criteria were age 30 years or older, diagnosis of PD [based on the presence of one of the following: (1) asymmetrical resting tremor or (2) asymmetrical bradykinesia or (3) at least two of either of resting tremor, bradykinesia, and rigidity], as well as a disease duration of one to 24 months, Hoehn and Yahr (H&Y) stage of 1–2, and presence of striatal dopamine transporter deficit on ^123^I-ioflupane SPECT imaging (DaTSCAN). The study was approved by the institutional review board at each site, and participants provided written informed consent. We obtained data from the PPMI database in compliance with the PPMI Data Use Agreement on 2nd of December 2019.

### Clinical evaluation

Data extracted from the PPMI database included demographics, age at onset, disease duration, as well as motor and non-motor symptom measures such as H&Y staging, Movement Disorder Society-Unified Parkinson’s Disease Rating Scale (MDS-UPDRS) (including Part I-Non-Motor Aspects of Experiences of Daily Living, Part II-Motor Aspects of Experiences of Daily Living, and Part III-Motor Examination) [23], SCOPA-Autonomic (SCOPA-AUT) [24], Montreal Cognitive Assessment (MoCA) [25], University of Pennsylvania Smell Identification Test (UPSIT), Geriatric Depression Scale (GDS), State-Trait Anxiety Inventory (STAI), and REM Behavior Sleep Disorder questionnaire (RBDQuest). Neuropsychological tests included Letter–Number Sequencing (working memory), Symbol-Digit Modalities Test (processing speed), animal fluency test (language/semantic fluency), Benton Judgment of Line Orientation 15-item (visuospatial functioning), and Hopkins Verbal Learning Test-Revised (HVLT, learning/immediate verbal memory and delayed verbal recall). ^123^I-FP-CIT striatal binding ratios, and cerebrospinal fluid (CSF) measures, specifically amyloid β 1–42 (Aβ_1-42_), total tau, and total α-synuclein were also extracted. At a follow-up of up to 6 years, patients were categorized as having normal cognition or cognitive impairment according to the PPMI protocol [22], Cognitive impairment was defined as scores on two or more of the HVLT total recall, HVLT recognition discrimination, Benton Judgment of Line Orientation, Letter–Number Sequencing, semantic (animal) fluency test, or Symbol-Digit Modalities Test of more than 1.5 standard deviations below normal, regardless of the presence of functional impairment due to cognitive dysfunction. The cognitive categorization in PPMI was implemented at a later stage, meaning that many patients only have cognitive categorization from 2-year follow-up visit onwards. Since this information was not available at earlier time points, patients with at least two cognitive tests of more than 1.5 standard deviations below normal at baseline were considered as “suspected MCI”, to reflect the lower level of confidence in this classification [26]. At baseline, patients were grouped into TD subtype and PIGD subtype, based on the MDS-UPDRS scores [5].

### Statistical analysis

Continuous variables were expressed as mean ± standard deviation, with between-group comparisons performed by one-way ANOVA or Mann–Whitney *U* test for normally or non-normally distributed variables, respectively. Categorical variables were expressed as proportions and compared using Pearson’s *χ*^2^ test. Patients categorized having an indeterminate subtype were excluded from the analysis. Cox survival analyses were performed including, as predictors, motor subtype (TD versus PIGD), individual MDS-UPDRS items defining PIGD phenotype (Part II: item 12 “Balance and Walking” and item 13 “Freezing”, Part III: item 10 “Postural Instability” and 12 “Gait”), known predictors of cognitive impairment (age, sex, years of educations, MDS-UPDRS Part III, RBDQuest, CSF Aβ42, UPSIT, and ^123^I-FP-CIT caudate uptake) [17] as well as anticholinergic burden [27] at univariate and multivariate levels. MDS-UPDRS Part III item 11 “Freezing of gait” was not included in the analysis as only three patients had this feature at baseline. The first occurrence of cognitive impairment at follow-up was used as the time-to-event in all Cox models. For these analyses, only patients with full cognitive testing on visits following baseline and without missing covariates were included. As the majority of patients’ first cognitive categorizations were made at the 2-year follow-up visit, there were 25 patients with MCI or PD dementia at their first categorization (up to the second year). As such, it is not possible to rule out that these patients did not already meet the criteria for MCI at baseline [26]. Thus, the Cox regression was repeated in a more restricted sample to assess whether postural instability was predictive of incident cognitive decline and did not depend on patients who may have had cognitively deteriorated at an earlier disease stage. In the repeated analysis, the criteria for “suspected MCI” (defined above) were applied at the baseline visit, and only participants without suspected MCI were included. Finally, Kaplan–Meier estimate and curve was generated, and comparisons were made using the log-rank (Mantel–Cox) test. Statistical analyses were performed using the Statistical Package for the Social Sciences (SPSS), version 25.0 (IBM Corp., Armonk, NY, USA). A *p* value of < 0.05 was considered statistically significant and Benjamini–Hochberg procedure was used to correct in case of multiple testing.

## Results

At baseline, 76 (18%) patients were classified having a PIGD subtype, 299 (71%) a TD subtype and 47 (11%) an indeterminate subtype. PIGD subtype patients had worse scores in activities of daily life (MDS-UPDRS Part II; *p* < 0.001) and H&Y scale (*p* = 0.023) compared with patients with TD subtype, and had a higher score of non-motor symptoms (MDS-UPDRS Part I; *p* = 0.008), specifically with higher anxiety (*p* < 0.004) and depression scores (*p* = 0.008) (Table 1). No other demographic or clinical differences were found between the groups. No differences were found in the detailed neuropsychological assessment, imaging, and CSF biomarkers.

**Table 1:** Demographic and clinical features associated with TD/PIGD classification in patients with de novo Parkinson’s disease

	TD (*n* = 299)	PIGD (*n* = 76)	*p* value
Demographic variables			
Age	61.89 ± 9.47	61.91 ± 9.23	0.988
Sex (male %)	66.6%	61.8%	0.440
Disease duration, months	6.76 ± 6.64	6.22 ± 5.48	0.841
Age of onset, years	59.75 ± 9.77	60.30 ± 9.54	0.572
Age of diagnosis, years	61.33 ± 9.44	61.40 ± 9.22	0.958
Clinical variables			
MDS-UPDRS Part I	5.17 ± 3.80	6.93 ± 4.75	0.008*
MDS-UPDRS Part II	5.27 ± 3.88	7.42 ± 4.57	0.00082*
MDS-UPDRS Part III	21.14 ± 9.042	21.00 ± 8.38	0.728
H&Y stage 1, 2, and 3 (%)	46.5, 53.5, 0	39.5, 57.9, 2.6	0.0234*
UPSIT	22.16 ± 7.86	22.50 ± 9.43	0.782
SCOPA-AUT	9.31 ± 6.315	9.59 ± 5.48	0.565
RBDSQ	4.02 ± 2.64	4.46 ± 2.94	0.480
STAI	63.74 ± 18.12	70.58 ± 17.49	0.0045*
GDS	2.06 ± 2.24	3.24 ± 2.87	0.0078*
DaTSCAN			
Mean caudate	2.01 ± 0.53	1.89 ± .63	0.067
Mean putamen	0.83 ± 0.27	0.79 ± 0.32	0.063
Mean striatum	1.42 ± 0.37	1.34 ± 0.45	0.079
Neuropsychological assessment			
MoCA	27.08 ± 2.34	27.15 ± 2.43	0.799
Benton judgment of line orientation	12.76 ± 2.13	12.61 ± 2.10	0.447
Symbol digit modalities score	41.16 ± 9.83	40.58 ± 10.00	0.771
Semantic fluency total score	48.35 ± 11.43	49.46 ± 13.41	0.493
Letter number sequencing raw score	10.55 ± 2.67	10.50 ± 2.73	0.842
HVLT immediate/total recall	24.46 ± 5.09	24.20 ± 4.888	0.829
HVLT delayed recall	8.42 ± 2.48	8.03 ± 2.79	0.362
HVLT delayed recognition	11.18 ± 1.30	11.14 ± 1.05	0.399
HVLT false alarms	1.30 ± 1.41	1.04 ± 0.93	0.493
HVLT recognition discrimination	9.51 ± 2.87	10.07 ± 1.41	0.877
HVLT retention	0.86 ± .197	0.82 ± 0.23	0.206
CSF variables			
CSF ABeta1-42 (pg/mL)	923.23 ± 427.65	888.01 ± 389.55	0.445
CSF total synuclein (pg/mL)	1540.13 ± 682.46	1440.89 ± 615.12	0.220
CSF total tau (pg/mL)	171.84 ± 58.99	167.30 ± 53.06	0.660

Data are presented as mean ± SD or percentage

*TD* tremor-dominant, *PIGD* postural instability and gait difficulties, *MDS-UPDRS* Movement Disorders Society-Unified Parkinson’s Disease Rating Scale, *UPSIT* University of Pennsylvania Smell Identification Test, *SCOPA-AUT* SCales for Outcomes in Parkinson’s disease Autonomic, *RBDSQ* REM Sleep Behavior Disorder Questionnaire Score, *STAI* State-Trait Anxiety Inventory; *GSD* Geriatric Depression Scale, *MoCA* Montreal cognitive assessment, *HVLT* Hopkins Verbal Learning Test, *CSF* cerebrospinal fluid

*Significant *p* values after Benjamini–Hochberg correction for multiple testing

**Fig. 1:**
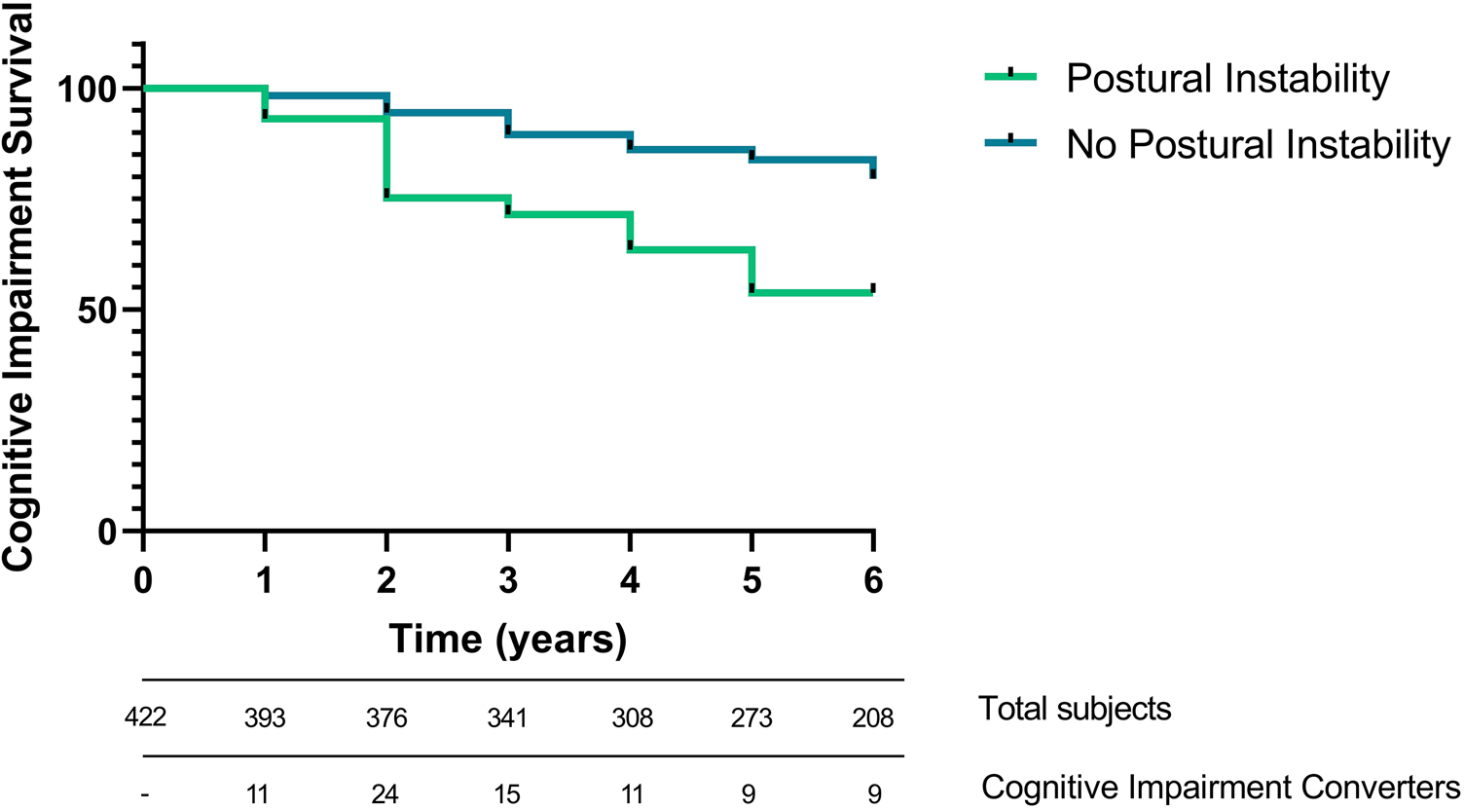
The risk of development of cognitive impairment in patients with postural instability (defined as UPDRS 3.12 item ≥ 1) in a Kaplan–Meier survival estimates plot (Log-Rank Mantel–Cox, *χ*^2^ 9.607, *p* = 0.002)

During a median follow-up of 5 years (IQ range, 3–6 years), 79 patients developed cognitive impairment. 36.3% of patients with postural instability at baseline (MDS-UPDRS 3.12 item ≥ 1) developed cognitive impairment, while 18.3% of patient without postural instability at baseline developed cognitive impairment over a median follow-up of 5 years. Kaplan–Meier curve and log-rank (Mantel-Cox) test showed that PD patients with postural instability (MDS-UPDRS 3.12 item ≥ 1) have shorter cognitive impairment-free survival times over a median follow-up of 5 years when compared to the PD patients without Postural Instability (Log-Rank 9.607, *p* = 0.002, Fig. 1). In the univariate Cox proportional-hazards models, conversion to cognitive impairment from normal cognition was not associated with motor subtype [PIGD versus TD subtype; hazard ratio (HR) 1.395; 95% Confidence Interval (CI) 0.804–2.300; *p* = 0.252, Table 2], gait score (MDS-UPDRS Part II. item 10 ≥ 1; HR = 1.036; 95%CI: 0.666–1.616; *p* = 0.870), “Walking and Balances” (MDS-UPDRS Part II item 12 ≥ 1; HR = 1.063; 95%CI: 0.670–1.685; *p* = 0.372), or “Freezing” (MDS-UPDRS Part II. item 13 ≥ 1; HR = 0.500; 95%CI: 0.123–2.035; *p* = 0.372). Postural instability (MDS-UPDRS III. item 12 ≥ 1) was a significant predictor of cognitive impairment in both the univariate (HR = 2.510; 95%CI: 1.356–4.646; *p* = 0.003) and the multivariate model (HR = 2.045; 95%CI: 1.068–3.918; *p* = 0.031), the latter including known predictors of cognitive impairment (age, years of education, total MDS-UPDRS III score, RBDSQ, CSF Aβ42, UPSIT, and mean caudate dopaminergic uptake). When restricting this analysis to only patients without “suspected MCI” at baseline, postural instability remained a significant predictor of cognitive impairment at both univariate (HR = 3.222; 95%CI: 1.498–6.931; *p* = 0.003, Table 3) and multivariate levels (HR = 2.573; 95%CI: 1.110–6.010; *p* = 0.029).

**Table 2:** Results of the Cox proportional-hazards analysis for the predictors of cognitive impairment

Variables	Univariable analysis		Multivariable analysis	
	HR (95% CI)	*p* value	HR (95% CI)	*p* value
Motor subtype (PIGD versus TD)	1.359 (0.804–2.300)	0.252	–	–
Walking and balance^a^	1.063 (0.670–1.685)	0.372	–	–
Freezing^a*^	0.500 (0.123–2.035)	0.333	–	–
Gait^b^	1.038 (0.666–1.616)	0.870	–	–
Postural instability^b^	2.510 (1.356–4.646)	0.003	2.045 (1.068–3.918)	0.031
Age	1.067 (1.039–1094)	< 0.001	1.053 (1.023–1.084)	0.001
Genre	1.646 (0.983–2.758)	0.058	0.922 (0.529–1.610)	0.776
Education (years)	0.933 (0.864–1.008)	0.077	0.925 (0.855–1.002)	0.056
MDS-UPDRS III^c^	1.038 (1.014–1.063)	0.002	1.029 (1.002–1.057)	0.034
RBDSQ	2.267 (1.456–3.531)	< 0.001	1.783 (1.120–2.839)	0.015
CSF Aβ42 (log)	0.081 (0.024–0.274)	< 0.001	0.096 (0.027–0.339)	< 0.001
UPSIT	0.930 (0.904–0.957)	< 0.001	0.957 (0.928–0.986)	0.05
Mean caudate uptake	0.366 (0.237–0.565)	< 0.001	0.561 (0.349–0.901)	0.015
ACB score	1.359 (0.870–2.122)	0.177	–	–

*MDS-UPDRS* Unified Parkinson’s Disease Rating Scale, *MoCA* Montreal cognitive assessment, *RBDSQ* REM Sleep Behavior Disorder Questionnaire Score, *UPSIT* University of Pennsylvania Smell Identification Test, *CSF* cerebrospinal fluid, *Aβ42* amyloid β 1–42

^a^Based on UPDRS part II items

^b^Based on UPDRS part III items

^c^To avoid collinearity, the items relative to “Postural Instability”, “Freezing”, and “Gait” were excluded by the UPDRS 3 total score

**Table 3:** Results of the Cox proportional-hazards analysis for the predictors of cognitive impairment

Variables	Univariable analysis		Multivariable analysis	
	HR (95% CI)	*p* value	HR (95% CI)	*p* value
Motor subtype (PIGD versus TD)	0.813 (0.412–1.605)	0.551	–	–
Walking and balance^a^	1.077 (0.579–2.003)	0.814	–	–
Freezing^a*^	0.047 (0.001–27.792)	0.347	–	–
Gait^b^	0.841 (0.469–1.508)	0.584	–	–
Postural instability^b^	3.222 (1.498–6.931)	0.003	2.573 (1.110–6.010)	0.029
Age	1.084 (1.046–1.123)	< 0.001	1.075 (1.032–1.120)	< 0.001
Genre	0.659 (0.341–1.277)	0.217	–	–
Education (years)	0.924 (0.828–1.031)	0.158	0.939 (0.838–1.052)	0.279
MDS-UPDRS III^c^	1.030 (0.997–1.063)	0.074	1.044 (1.015–1.074)	0.003
RBDSQ	1.983 (1.105–3.557)	0.022	1.157 (0.846–2.943)	0.152
CSF Aβ42 (log)	0.108 (0.021–0.543)	0.108	0.131 (0.025–0.702)	0.018
UPSIT	0.931 (0.897–0.966)	< 0.001	0.955 (0.920–0.992)	0.018
Mean caudate uptake	0.238 (0.131–0.434)	< 0.001	0.356 (0.186–0.681)	0.002
ACB score	1.268 (0.699–2-304)	0.435	–	–

PD patients with “Suspected MCI” at baseline have been excluded from this analysis

*MDS-UPDRS* Unified Parkinson’s Disease Rating Scale, *MoCA* Montreal cognitive assessment, *RBDSQ* REM Sleep Behavior Disorder Questionnaire Score, *UPSIT* University of Pennsylvania Smell Identification Test, *CSF* cerebrospinal fluid, *Aβ42* amyloid β 1–42

^a^Based on UPDRS part II items

^b^Based on UPDRS part III items

^c^To avoid the collinearity, the items relative to “Postural Instability”, “Freezing”, and “Gait” were excluded by the UPDRS 3 total score

## Discussion

Our findings suggest that motor subtyping based on TD/PIGD classification may not be relevant for the prediction of cognitive impairment in de novo PD patients as has been proposed in the previous studies. We would like to propose that postural instability alone, as assessed by the MDS-UPDRS Part III, may serve as a possible indicator for the risk of developing cognitive impairment.

The distinction between TD and PIGD is one of the most cited forms of PD subtyping. This motor subtype approach was conceptualized by Jankovic et al. [3] in their retrospective review of the DATATOP trial, using the ratio of tremor-related items on the UPDRS to PIGD-related items. This classification system was then adapted for the MDS-UPDRS motor scale [5] and has been extensively adopted in clinical trials and observational studies. The PIGD subtype has been associated with a higher burden of non-motor features than the TD subtype, particularly affective symptoms [8, 28]. This is in line with our baseline results, as we found that patients classified as PIGD subtype had a higher burden of non-motor symptoms, including worse anxiety and depression. PIGD has also been associated with the development of cognitive impairment and several studies have highlighted the relationship between PIGD severity and amyloid-β pathology through CSF measurement [29] or Positron Emission Tomography (PET) imaging [13]. However, some studies have failed to demonstrate that this subtype was associated with the development of PD cognitive milestones [17]. Others have found that the transition between the TD to the PIGD phenotype, and not the baseline characterization, was able to predict the risk of dementia [30]. Simuni et al. [19] explored the stability of the TD/PIGD classification in an early untreated PD population, reporting that patients shifted bidirectionally between phenotypic subtypes as the disease progressed over the first year. Furthermore, many patients who had TD/PIGD ratios that fell close to the indeterminate range were eliminated from this categorization and further analysis. Taking into consideration the above-mentioned issues, new arguments suggest that these motor phenotypes should not be assessed as discrete subtypes, but rather as a multi-dimensional continuum [31]. Our results showed that postural instability in itself may be an independent predictor of cognitive impairment in de novo PD patients, suggesting that disentangling the PIGD classification into individual items could be useful for predicting long-term development of PD cognitive milestones, especially in the very early phases of PD, when patients’ motor features have not yet “matured” [19]. As such, it may be argued that motor subtyping in de novo PD cannot be reliably performed and should not be used in predictive studies, and that individual MDS-UPDRS items may prove more useful as predictors for later outcome. Addition of non-motor subtyping, as has been proposed based on cluster analysis [32, 33] as well as clinical phenotyping [34], should also be considered.

Postural instability is a cardinal feature in PD [1] and is a key staging marker on the modified Hoehn and Yahr scale [35] marking the transition from early, mild disease to late, severe disease. Being relatively uncommon in the early stage of the disease course, one could hypothesise that postural instability might be a more sensitive predictor of worse long-term outcomes, such as cognitive impairment. Postural instability is usually clinically documented using the quick and easy retropulsion test (Pull Test, MDS-UPDRS Part III item 12). Postural and balance deficits in PD may result from lesions to both dopaminergic and non-dopaminergic nuclei [36]. The impaired cholinergic transmission in the pedunculopontine nucleus (PPN), known to degenerate in PD [37, 38], has shown to be implicated in the occurrence of postural instability [39], impinging on the attentional control of posture and detection of movement errors [40]. Interestingly, cholinergic perturbations have also been robustly associated with cognitive impairment in PD [41].

On the other hand, our results showed that gait difficulties do not predict cognitive impairment in early PD. Of note, gait difficulties in PD are multifactorial in nature and common comorbidities like peripheral neuropathy and osteo-arthritis, can further impair this motor feature [42]. To some extent, every aspect of gait evaluated through the MDS-UPDRS item has a substantial dopaminergic-related component (“velocity and step length stride amplitude, stride speed, height of foot lift, heel strike during walking, turning, and arm swing”) that can be directly related to rigidity or bradykinesia, especially during the early stage. These observations indicate that gait difficulties, as conventionally evaluated, are less sensitive than postural instability in identifying early PD patients with a more extensive (extra-nigral, non-dopaminergic) underlying pathology and thus more prone to develop cognitive impairment.

Limitations of our study include the use of subjective outcome measures characterized by limited reliability compared to objective device-based motor data. Nevertheless, the MDS-UPDRS represents a validated and easy-to-use tool that can be used in clinical settings. Second, we did not evaluate how the severity of postural instability was correlated to the longitudinal outcomes. Nevertheless, since this motor feature is less common in the early stage of PD, in our analyses, we focused on its presence or absence as a categorical variable, to define a subgroup of de novo patients experiencing this symptom even with a mild severity. Finally, we should mention that PPMI neuropsychological battery only allows a Level I classification of cognitive impairment that is less sensible and more prone to false-negative findings compared to the Level II classification, which requires two tests within each of the five cognitive domains (i.e., attention and working memory, executive, language, memory, and visuospatial) [43].

In conclusion, our results suggest that postural instability, but not gait difficulties or the TD/PIGD motor subtyping, may be appropriate for the prognostication of cognitive impairment in early de novo PD and emphasize the need for greater vigilance in this subgroup of patients.

## References

[1] Kalia LV, Lang AE (2015). Parkinson’s disease. Lancet.

[2] Zetusky WJ, Jankovic J, Pirozzolo FJ (1985). The heterogeneity of parkinson’s disease: clinical and prognostic implications. Neurology.

[3] Jankovic J, McDermott M, Carter J, Gauthier S, Goetz C, Golbe L (1990). Variable expression of Parkinson’s disease. a base-line analysis of the DATATOP cohort. Neurology.

[4] Aleksovski D, Miljkovic D, Bravi D, Antonini A (2018). Disease progression in Parkinson subtypes: the PPMI dataset. Neurol Sci.

[5] Stebbins GT, Goetz CG, Burn DJ, Jankovic J, Khoo TK, Tilley BC (2013). How to identify tremor dominant and postural instability/gait difficulty groups with the movement disorder society unified Parkinson’s disease rating scale: comparison with the unified Parkinson’s disease rating scale. Mov Disord.

[6] Thenganatt MA, Jankovic J (2014). Parkinson disease subtypes. JAMA Neurol.

[7] Van Der Heeden JF, Marinus J, Martinez-Martin P, Rodriguez-Blazquez C, Geraedts VJ, Van Hilten JJ (2016). Postural instability and gait are associated with severity and prognosis of Parkinson disease. Neurology.

[8] Marras C, Chaudhuri KR (2016). Nonmotor series: nonmotor features of Parkinson’ s disease subtypes. Mov Disord.

[9] Choi SM, Kim BC, Cho BH, Kang KW, Choi KH, Kim JT (2018). Comparison of two motor subtype classifications in de novo Parkinson's disease. Parkinsonism Relat Disord.

[10] Burn DJ, Rowan EN, Allan LM, Molloy S, O'Brien JT, McKeith IG (2006). Motor subtype and cognitive decline in Parkinson's disease, Parkinson's disease with dementia, and dementia with Lewy bodies. J Neurol Neurosurg Psychiatry.

[11] Williams-Gray CH, Foltynie T, Brayne CEG, Robbins TW, Barker RA (2007). Evolution of cognitive dysfunction in an incident Parkinson's disease cohort. Brain.

[12] Marras C (2015). Subtypes of Parkinson's disease: state of the field and future directions. Curr Opin Neurol.

[13] Müller MLTM, Frey KA, Petrou M, Kotagal V, Koeppe RA, Albin RL (2013). β-amyloid and postural instability and gait difficulty in Parkinson's disease at risk for dementia. Mov Disord.

[14] Kang JH, Irwin DJ, Chen-Plotkin AS, Siderowf A, Caspell C, Coffey CS (2013). Association of cerebrospinal fluid β-amyloid 1–42, t-tau, p-tau 181, and α-synuclein levels with clinical features of drug-naive patients with early parkinson disease. JAMA Neurol.

[15] Lee JW, Song YS, Kim H, Ku BD, Lee WW (2019). Alteration of tremor dominant and postural instability gait difficulty subtypes during the progression of Parkinson's disease: analysis of the PPMI cohort. Front Neurol.

[16] Selikhova M, Williams DR, Kempster PA, Holton JL, Revesz T, Lees AJ (2009). A clinico-pathological study of subtypes in Parkinson’s disease. Brain.

[17] Schrag A, Siddiqui UF, Anastasiou Z, Weintraub D, Schott JM (2017). Clinical variables and biomarkers in prediction of cognitive impairment in patients with newly diagnosed Parkinson’s disease: a cohort study. Lancet Neurol.

[18] Johnson AR, Bucks RS, Kane RT, Thomas MG, Gasson N, Loftus AM (2016). Motor subtype as a predictor of future working memory performance in idiopathic Parkinson’s disease. PLoS ONE.

[19] Simuni T, Caspell-Garcia C, Coffey C, Lasch S, Tanner C, Marek K (2016). How stable are Parkinson's disease subtypes in de novo patients: Analysis of the PPMI cohort?. Parkinsonism Relat Disord.

[20] Konno T, Deutschländer A, Heckman MG, Ossi M, Vargas ER, Strongosky AJ (2018). Comparison of clinical features among Parkinson's disease subtypes: a large retrospective study in a single center. J Neurol Sci.

[21] Factor SA, Kyle Steenland N, Higgins DS, Molho ES, Kay DM, Montimurro J (2011). Postural instability/gait disturbance in Parkinson's disease has distinct subtypes: an exploratory analysis. J Neurol Neurosurg Psychiatry.

[22] Marek K, Jennings D, Lasch S, Siderowf A, Tanner C, Simuni T (2011). The parkinson progression marker initiative (PPMI). Prog Neurobiol.

[23] Goetz CG, Tilley BC, Shaftman SR, Stebbins GT, Fahn S, Martinez-Martin P (2008). Movement Disorder Society-sponsored revision of the Unified Parkinson’s Disease Rating Scale (MDS-UPDRS): scale presentation and clinimetric testing results. Mov Disord.

[24] Visser M, Marinus J, Stiggelbout AM, Van Hilten JJ (2004). Assessment of autonomic dysfunction in Parkinson’s disease: the SCOPA-AUT. Mov Disord.

[25] Nasreddine ZS, Phillips NA, Bedirian V, Charbonneau S, Whitehead V, Collin I (2005). The Montreal Cognitive Assessment, MoCA: a brief screening tool for mild cognitive impairment. J Am Geriatr Soc.

[26] Ray NJ, Bradburn S, Murgatroyd C, Toseeb U, Mir P, Kountouriotis GK (2018). In vivo cholinergic basal forebrain atrophy predicts cognitive decline in de novo Parkinson's disease. Brain.

[27] Weintraub D, Caspell-Garcia C, Simuni T, Cho HR, Coffey CS, Aarsland D (2020). Neuropsychiatric symptoms and cognitive abilities over the initial quinquennium of Parkinson disease. Ann Clin Transl Neurol.

[28] Reijnders JSAM, Ehrt U, Lousberg R, Aarsland D, Leentjens AFG (2009). The association between motor subtypes and psychopathology in Parkinson's disease. Parkinsonism Relat Disord.

[29] Alves G, Pedersen KF, Bloem BR, Blennow K, Zetterberg H, Borm GF (2013). Cerebrospinal fluid amyloid-β and phenotypic heterogeneity in de novo Parkinson’s disease. J Neurol Neurosurg Psychiatry.

[30] Alves G, Larsen JP, Emre M, Wentzel-Larsen T, Aarsland D (2006). Changes in motor subtype and risk for incident dementia in Parkinson’s disease. Mov Disord.

[31] Kotagal V (2016). Is PIGD a legitimate motor subtype in Parkinson disease?. Ann Clin Transl Neurol.

[32] Martinez-Martin P, Rojo-Abuín JM, Weintraub D, Chaudhuri KR, Rodriguez-Blázquez C, Rizos A (2020). Factor analysis and clustering of the movement disorder society-non-motor rating scale. Mov Disord.

[33] Mu J, Chaudhuri KR, Bielza C, de Pedro-Cuesta J, Larrañaga P, Martinez-Martin P (2017). Parkinson’s disease subtypes identified from cluster analysis of motor and non-motor symptoms. Front Aging Neurosci.

[34] Sauerbier A, Jenner P, Todorova A, Chaudhuri KR (2016). Non motor subtypes and Parkinson’s disease. Parkinsonism Relat Disord.

[35] Goetz CG, Poewe W, Rascol O, Sampaio C, Stebbins GT, Counsell C (2004). Movement Disorder Society Task Force report on the Hoehn and Yahr staging scale: status and recommendations. Mov Disord.

[36] Bloem BR, Beckley DJ, Gert Van Dijk J, Zwinderman AH, Remler MP, Roos RAC (1996). Influence of dopaminergic medication on automatic postural responses and balance impairment in Parkinson’s disease. Mov Disord.

[37] Bohnen NI, Müller MLTM, Koeppe RA, Studenski SA, Kilbourn MA, Frey KA (2009). History of falls in Parkinson disease is associated with reduced cholinergic activity. Neurology.

[38] Welter ML, Demain A, Ewenczyk C, Czernecki V, Lau B, El Helou A (2015). PPNa-DBS for gait and balance disorders in Parkinson’s disease: a double-blind, randomised study. J Neurol.

[39] Müller MLTM, Bohnen NI (2013). Cholinergic dysfunction in Parkinson’s disease. Curr Neurol Neurosci Rep.

[40] Sarter M, Albin RL, Kucinski A, Lustig C (2014). Where attention falls: Increased risk of falls from the converging impact of cortical cholinergic and midbrain dopamine loss on striatal function. Exp Neurol.

[41] Aarsland D, Creese B, Politis M, Chaudhuri KR, Dominic H, Weintraub D (2017). Cognitive decline in Parkinson disease. Nat Rev Neurol.

[42] Mirelman A, Bonato P, Camicioli R, Ellis TD, Giladi N, Hamilton JL (2019). Gait impairments in Parkinson's disease. Lancet Neurol.

[43] Litvan I, Goldman JG, Tröster AI, Schmand BA, Weintraub D, Petersen RC (2012). Diagnostic criteria for mild cognitive impairment in Parkinson's disease: movement Disorder Society Task Force guidelines. Mov Disord.

